# Practice what you preach: Importance of veterinarian involvement in zoonotic disease prevention – A Michigan focus

**DOI:** 10.18849/ve.v7i2.512

**Published:** 2022-04-27

**Authors:** Stephanie J.S. Baiyasi+, Rubén Juárez+, Jodi Brookins-Fisher+, Jeff N. Inungu+, Thomas M. Gehring+, Zigmond A. Kozicki+

**Keywords:** ANIMAL DISEASE, DISEASE PREVENTION, ONE HEALTH, ZOONOSES, ZOONOTIC DISEASE PREVENTION

## Abstract

**Objective:**

Determine the extent to which practicing veterinarians in Michigan, USA engaged in commonly recommended practices for the prevention of zoonotic diseases (ZDs).

**Background:**

Follow-up to Lipton et al. (2008) Washington State study.

**Methods:**

Online survey link was emailed February 2020 to 3,410 Michigan licensed veterinarians practicing clinical medicine with emails on file with Michigan Licensing and Regulatory Affairs.

**Result:**

402 veterinarians responded. A high proportion (161/214 [75%]) of respondents agreed it was very important for veterinarians to advise clients about the potential for ZD, yet only 34% (74/215) reported they had initiated discussions about ZDs with clients on a daily basis, although 64% (137/214) indicated they had client educational materials on ZDs available in their practices. Nearly 62% (47/76) of veterinarians who obtained their degree after 2010 were likely to eat / drink in animal handling areas as compared to only 33% (18/54) of those who graduated before 1989. Over 30% of respondents (64/210) indicated there were no written infection control guidelines for staff members in the practice, and 28% (60/214) reported having been infected with a ZD in practice.

**Conclusion:**

Veterinarians appreciate their important role in ZD prevention and welcome increased communication between human and veterinary medicine plus assistance from public health agencies regarding ZD prevention. Communication / coordination / collaboration among human medicine / animal medicine / environmental health (i.e., One Health) is necessary to protect the public’s health from zoonoses.

## INTRODUCTION

Zoonoses are infectious diseases that are transmitted between species from animals to humans (or from humans to animals) (Minnesota Department of Health, 2019). Of the 1,461 infectious diseases recognised to occur in humans, approximately 60% are caused by multihost pathogens, characterised by their movement across various species (Bidaisee & Macpherson, 2014). Of the total number of human diseases, 177 are regarded as emerging or reemerging zoonotic pathogens and are twice as likely to be in this category as are nonzoonotic pathogens (Woolhouse & Gowtage-Sequeria, 2005). The recent pandemic of COVID-19 caused by SARS-CoV-2 (a coronavirus similar to the agent causing ‘severe acute respiratory syndrome [SARS]’) is an example of a zoonotic agent’s potential to disrupt human life (Khan et al., 2020).Exposure to and emergence of zoonotic pathogens can occur in various ways including companion animals (dogs, cats, exotic pets), recreationally (water sports, hunting, and the movement of animals for these and other sporting purposes), globalisation, tourism, livestock movement, and changed land use and urbanisation (Cutler et al., 2010). The general population, including most pet owners, are unaware that pets and other animals can carry infectious agents transmissible to people and are not familiar with methods to prevent zoonotic diseases (Lipton et al., 2008). Stull et al. (2012) found a need for accessible zoonotic disease information for the public and additional efforts to educate clientele were needed from veterinarians, physicians and public health personnel. This study examined the extent that veterinarians in Michigan engage in commonly recommended practices for the prevention of zoonotic diseases. 

## METHODS

**Study protocol:** The study was designed as a cross-sectional survey. A sample of veterinarians licensed in Michigan was selected from the Michigan Licensing and Regulatory Affairs (LARA) office per a Freedom of Information Act (FOIA) request. The original file included all Michigan licensed veterinarians and veterinary technicians (8,052 individuals), including those with and without email addresses as of February 2020. Only veterinarians with listed email addresses were included for the survey which produced a list of 3,434 emails. Duplicate emails were deleted and 3,410 participants were emailed a request to complete the survey. The study protocol was approved by the university institutional review board. 

**Survey Instrument:** The 40 question survey instrumenta for this research was modified from a previously developed and validated 31 item instrument (Lipton et al., 2008) that was used to determine the extent to which practicing veterinarians in King County, Washington, engaged in commonly recommended practices for the prevention of zoonotic diseases. This 40 question survey was implemented using commercially available softwareb. The survey link was emailed with an informed consent page to start the survey. Most questions were closed-ended and the survey took approximately 15 minutes to complete. The survey began by screening if veterinarians were in clinical practice. If not, they answered the final question of the survey. Those in clinical practice answered questions in five main themes.

**Themes:** The first theme ascertained demographic characteristics of the respondents, including the type of veterinary medical practice, years in clinical practice, average number of days worked per week, number and type of staff in the practice, gender of respondent, veterinary school attended and year of graduation, plus any advanced degrees or certifications. The second theme determined how often respondents had discussed zoonotic diseases with clients during the past year (including veterinarian-initiated and client-initiated discussions), how many cases of zoonotic disease in animals had been diagnosed in the past 5 years, and how often clients had described themselves or a family member as being at higher risk of infection (e.g. immunocompromised, pregnant, elderly, or having children < 6 years old) and asked about zoonotic diseases during the past year. This theme of questions included the likelihood of discussing zoonotic diseases if a client or client’s family member was known to be at higher risk of infection and the specific zoonotic diseases discussed with clients. Respondents were asked whether continuing education courses on zoonotic diseases were available and if they completed any in the last 3 years. The third theme addressed zoonotic disease discussions with client or non-client healthcare professionals (e.g. physicians, physician assistants, nurse practitioners, nurses), public health officials, or wildlife biologists during the past year. This theme also included respondents’ involvement with local and state public health agencies, including questions about how often they had contacted the local or state public health agency or the Michigan Department of Agriculture and Rural Development (MDARD), Animal Industry Division (State Veterinarian’s Office) to discuss reportable zoonotic diseases in animals during the past year; a self-assessment of respondents’ knowledge of which diseases they were required by state law to report to the Michigan Department of Health and Human Services (MDHHS); knowledge of who to contact about a suspected case or outbreak of zoonotic disease; and whether and how public health agencies could better assist with issues involving zoonotic diseases. Veterinarians that indicated public health agencies could better assist with issues involving zoonotic diseases were asked to choose one or more ways from a list of eight options and describe any other ways public health agencies could assist.The fourth theme addressed the importance of advising clients about zoonotic diseases, as well as the zoonotic diseases covered in and availability of educational materials for clients. Additionally, this theme covered whether respondents had ever been infected with a zoonotic disease in practice and what various infection control measures in practice (e.g. washing hands between clients, eating / drinking in animal handling areas, disinfecting exam table frequency, presence of written infection control guidelines) had been used. Respondents who indicated that they had been infected with a zoonotic disease were asked to indicate the disease of which they had been infected and whether the diagnosis had been medically confirmed.The final theme addressed the One Health initiative. This concept is defined as a collaborative, multisectoral, and transdisciplinary approach that involves working at the local, regional, national, and global levels, with the goal of achieving optimal health outcomes by recognising the interconnection between people, animals, plants, and their shared environment. Participants were asked to describe their familiarity with this initiative.

**Statistical Analysis:** Survey data were analysed with standard softwarec. Descriptive statistics (frequencies, range of scores, means, standard deviations) were utilised to describe the respondents in terms of their demographic and background characteristics, participation in zoonotic disease prevention activities, and communication about zoonotic disease prevention in the community. To assess the relationship of demographic variables (sex, year of graduation, years in practice, advanced degree) on zoonotic disease prevention practices, a series of Chi-square analyses were performed. In cases where respondents had missing data, analyses were conducted only on complete responses. Values of p < 0.05 were considered significant. Qualitative data were reviewed and coded by hand to identify common responses. Our research was approved by the Institutional Review Board at Central Michigan University (IRB # 2020-185). 

## RESULTS

General

Among the 402 respondents, 261 (64.9%) responded after the initial email request, 57 (14.2%) responded after the first reminder email, and 84 (20.9%) responded after the second reminder email. The 269 (66.9%) who self-identified as active veterinarians were directed to the remaining questions of the survey. A total of 215 participants completed at least 65% of the remaining survey and these responses were used in the study analysis. Power analysis indicated that our sample size (n = 215) corresponded to a 5% margin of error at a confidence level of 90% and a 6% margin of error at a confidence level of 95%.

Demographics

Most respondents (157/215 [73.0%]) practiced small animal medicine as either the sole practice type or in combination with other types (see Table 1). 44/215 (20.5%) respondents had two practice types and 21/215 (9.8%) had three or more practice types (see Table 2). Regarding where the veterinary degree was obtained, 145/215 respondents (67.4%) indicated Michigan State University / MSU (although ‘MSU’ could potentially indicate another out-of-state university), while 69/215 (32.1%) received their veterinary degree from another school, including 17/69 (24.6%) respondents who attended a foreign school. Female participants (154/215) comprised 71.6% of the sample, 59/215 were male (27.4%), and 2/215 (0.9%) respondents preferred not to disclose their gender. Of the 44 respondents who possessed advanced degrees or certifications, 8/44 (18.2%) were board certified (in dermatology, emergency and critical care, reproductive medicine, internal medicine, laboratory animal medicine, surgery, and / or veterinary behaviour), 5/44 (11.4%) had degrees in public health, and 3/44 (6.8%) had Master in Business Administration (MBA) degrees.

**Table 1:** Practice type of veterinarians in Michigan, who responded to a survey on zoonotic disease prevention practices.

Type of Veterinary Practice	No.	%
Small animal (dog / cat)	157	73.4%
Small animal emergency	31	14.5%
Exotic pets (pocket pets / reptiles / birds / fish, etc.)	29	13.6%
Livestock (cattle / sheep / pig, etc.)	22	10.3%
Mixed animal (small and large animal)	14	6.5%
Shelter medicine	13	6.1%
Equine	19	8.9%
Wildlife / zoo	8	3.7%
All animal species	1	0.5%
Aquatic only	1	0.5%
Government	1	0.5%
Laboratory animal	1	0.5%
Lab animal medicine / teaching / small animal	1	0.5%
Laboratory	1	0.5%
Poultry	1	0.5%
Regulatory medicine	1	0.5%
Relief	1	0.5%
Specialty, majority small animal with some large and exotics	1	0.5%
Wellness clinics	1	0.5%
Total Respondents*	215	100%

*Reflects total number of individuals responding; each respondent may have selected more than one practice type.

**Table 2:** Demographic characteristics of veterinarians in Michigan that responded to the survey

Practice type*	Frequency		Gender**				Years in practice						Advanced degree or certification*			
	No.	%	Male		Female		≤ 5		6–15		>15		No		Yes	
Small animal (Dog / Cat)	104	48.4	27	26%	77	74%	14	13.5%	23	22.1%	67	64.4%	84	81.6%	19	18.4%
Two practice types	44	20.5%	6	14%	37	86%	11	25%	16	36.4%	17	38.6%	34	77.3%	10	22.7%
Three or more practice types	21	9.8%	7	35%	13	65%	10	47.6%	6	28.6%	5	23.8%	20	95.2%	1	4.8%
Livestock (Cattle / Sheep / Pig, etc.)	13	6%	10	76.9%	3	23.1%	4	30.8%	3	23.1%	6	46.2%	11	84.6%	2	15.4%
Mixed animal (small and large animal)	9	4.2%	3	33.3%	6	66.7%	2	22.2%	4	44.4%	3	33.3%	6	66.7%	3	33.3%
Other	9	4.2%	1	11.1%	8	88.9%	2	22.2%	2	22.2%	5	55.6%	5	55.6%	4	44.%
Equine	6	2.8%	2	33.3%	4	66.7%	2	33.3%	1	16.7%	3	50%	4	66.7%	2	33.3%
Small animal emergency	5	2.3%			5	100%	2	40%	3	60%			5	100%		
Shelter medicine	3	1.4%	2	66.7%	1	33.3%	1	33.3%			2	66.7%	1	33.3%	2	66.7%
Did not specify	1	0.5%	1	100%							1	100%			1	100%
Total	215		59		154		48		58		109		170		44	

*Responses were missing for one individual.

**Responses were missing for two individuals.

Veterinarian zoonotic disease discussions

When asked about their discussions about zoonotic diseases with clients, health care professionals, public health officials, and wildlife biologists, 209/215 (97.2%) respondents reported that they had initiated discussions about zoonotic diseases with clients in the past year, 151/209 (72.2%) did so on a daily or weekly basis, while 147/209 (70.3%) reported that clients initiated discussion about zoonotic diseases monthly or less frequently (see Table 3). 114/215 (53.0%) participants indicated they were much more likely to discuss zoonotic diseases if they knew that a client or the client’s family members were at a risk of infection. Only 2/215 (0.9%) of surveyed veterinarians indicated that on a daily basis, clients described themselves or their family members as having a higher risk of infection and asked about zoonotic diseases. 113/215 (52.6%) respondents indicated that at-risk clients did this only occasionally, and 58/215 (26.9%) reported at-risk clients never asking the veterinarian about zoonotic diseases. 191/215 (88.8%) respondents indicated discussing zoonotic diseases with health care professionals (e.g. physicians, physician assistants, nurse practitioners, nurses) at least once during the past year. 93/215 (43.3%) respondents indicated they had discussed zoonotic diseases with public health officials at least once during the past year, while 43/215 (20%) respondents indicated they had discussed zoonotic diseases with wildlife biologists at least once during the past year.

**Table 3:** Zoonotic disease discussion frequency between Michigan veterinarians and clients.

Frequency	Veterinarian initiated		Client initiated	
Daily	74	35.4%	15	7.2%
Weekly	77	36.8%	47	22.5%
Monthly	28	13.4%	38	18.2%
Occasionally	29	13.9%	97	46.4%
Never	1	0.5%	12	5.7%
Total	209		209	

Data are given as number of respondents and percentages. There were six respondents who indicated that they do not have any client contact in their type of clinical practice.

Zoonotic disease topics

In the survey, respondents were given a list of 35 zoonotic disease topics and asked to indicate the topics they had discussed with their clients. The 10 most frequently discussed zoonotic disease topics in the order of most to least discussed were internal parasitism, external parasitism, rabies, leptospirosis, dermatophytosis, giardiasis, Lyme disease, animal bite prevention, salmonellosis, and feeding raw food diets (see Table 4). 73/208 (35.1%) respondents indicated never discussing visceral and ocular larval migrans with their clients, and 114/209 (54.5%) indicated that they never discussed *Baylisascaris* *spp*. (raccoon roundworm) with their clients. By contrast, 201/212 (94.8%) respondents indicated that they discussed internal parasitism in general with their clients. Veterinarians were more likely to initiate discussions about leptospirosis, giardiasis, rabies, internal parasitism, and salmonellosis, whereas clients were more likely to initiate discussion about dermatophytosis / ringworm, feeding raw food diets, animal bite prevention, toxoplasmosis, and Lyme disease.

**Table 4:** Specific zoonotic diseases or topics discussed with clients, as reported by veterinarians in Michigan.

Ever discussed						Never discussed		Total sum of responses on disease / topic	
Disease or topic	Veterinarian initiated		Client initiated		Either initiated				
Leptospirosis	178	83.6%	7	3.3%	12	5.6%	16	7.5%	213
Giardia	159	76.4%	5	2.4%	19	9.1%	25	12%	208
Rabies	154	73.3%	8	3.8%	36	17.1%	12	5.7%	210
Control of internal parasites	151	71.2%	6	2.8%	44	20.8%	11	5.2%	212
Salmonella	137	65.6%	5	2.4%	24	11.5%	43	20.6%	209
Scabies	130	62.5%	7	3.4%	17	8.2%	54	26%	208
Visceral or ocular larval migrans	127	61.1%	1	0.5%	7	3.4%	73	35.1%	208
Raw meat ‘bones and raw food’ (BARF) diets	117	56%	26	12.4%	18	8.6%	48	23%	209
Control of external parasites	116	55.2%	8	3.8%	74	35.2%	12	5.7%	210
Lyme disease	116	55.2%	21	10%	43	20.5%	30	14.3%	210
Enteric infections in general	114	54.8%	13	6.3%	30	14.4%	51	24.5%	208
Dermatophytosis / ringworm	112	52.8%	29	13.7%	54	25.5%	17	8%	212
Animal bite prevention	110	52.1%	22	10.4%	40	19%	39	18.5%	211
Toxoplasmosis	110	52.6%	22	10.5%	24	11.5%	53	25.9%	209
E. coli	109	52.9%	6	2.9%	25	12.1%	66	32%	206
Methicillin-resistant Staphylococcus aureus (MRSA)	98	46.7%	18	8.6%	30	14.3%	64	30.5%	210
Cat scratch disease	93	44.5%	21	10.1%	26	12.4%	69	33%	209
Rocky Mt. spotted fever	86	41.8%	2	1%	6	2.9%	112	54.4%	206
Baylisascaris spp. (raccoon roundworm)	85	40.7%	5	2.4%	5	2.4%	114	54.6%	209
Cryptosporidium	73	35.3%	4	1.9%	10	4.8%	120	58%	207
Campylobacter	64	30.9%	3	1.5%	11	5.3%	129	62.3%	207
Brucellosis	63	30%	10	4.8%	6	2.9%	131	62.4%	210
West Nile virus	26	12.6%	12	5.8%	23	11.1%	146	70.5%	207
Eastern equine encephalitis	25	12%	15	7.2%	21	10.1%	147	70.7%	208
Chronic wasting disease	16	7.7%	15	7.2%	17	8.2%	160	76.9%	208
Tularemia	16	7.8%	0	0%	6	2.9%	184	89.3%	206
Plague	12	5.8%	0	0%	6	2.9%	189	91.3%	207
Psittacosis	12	5.8%	2	1%	4	1.9%	189	91.3%	207
Scrapie	11	5.3%	4	1.9%	7	3.4%	184	89.3%	206
Avian influenza (bird flu)	10	4.9%	18	8.7%	15	7.3%	163	79.1%	206
BSE (mad cow)	9	4.3%	9	4.3%	9	4.3%	181	87.0%	208
Lymphocytic choriomeningitis	8	3.9%	0	0%	9	4.4%	190	91.8%	207
Hantavirus	6	2.9%	0	0%	4	1.9%	196	95.2%	206
Monkey pox	5	2.4%	1	0.5%	5	2.4%	196	94.7%	207
Rat bite fever	3	1.5%	0	0%	6	2.9%	197	95.6%	206

Importance of zoonotic disease advising

161/214 (75.2%) respondents indicated they felt it was very important to advise clients about the potential for zoonotic disease; 35/214 (16.4%) indicated it was moderately important and 15/214 (7.0%) indicated it was somewhat important. 137/214 (64%) respondents indicated that they did have client educational materials on zoonotic diseases available in their practices. The five most common zoonotic diseases included in client educational materials available in practices that had such materials were leptospirosis, rabies, Lyme disease, intestinal parasitism, and external parasitism.

Continuing education

The next section on the survey asked participants about their own continuing education on zoonotic diseases. Of those who responded, 139/213 (65.3%) indicated they had attended continuing education courses on zoonotic diseases within the past 3 years, and 120/215 (55.8%) indicated they believed that continuing education courses on zoonotic diseases were regularly available.

Infection control practices

When asked about infection control practices, 184/211 (87.2%) respondents indicated they always or most of the time washed their hands between handling individual animals, and only 27/211 (12.8%) indicated that they sometimes, seldom, or never washed their hands between handling individual animals. Additionally, 165/206 (80.1%) respondents indicated that examination and treatment tables were always disinfected between patients, and only 9/205 (4.4%) respondents indicated that tables were seldom or never disinfected between patients. 82/210 (39.1%) respondents indicated that veterinarians or staff members ate or drank in animal handling areas daily, 72/210 (34.3%) indicated that veterinarians or staff members ate or drank in animal handling areas occasionally, and 37/210 (17.6%) indicated that veterinarians and staff members never ate or drank in animal handling areas. 106/210 (50.5%) respondents indicated there were written infection control guidelines for staff members in the practice. 64/210 (30.5%) respondents indicated there were no written infection control guidelines for staff members in the practice, and an additional 40/210 (19.1%) were unsure whether the practice had written infection control guidelines. It should be noted that these results were self-reported and therefore subject to potential bias.

Results (self-reported) suggested that those with less veterinarian experience were more likely to eat and drink in animal handling areas. Categorising respondent year of graduation (2010 or later, 2000–2009, 1990–1999, 1989 or earlier), a Chi-square test of independence was performed comparing the frequency of categorised year of graduation from veterinarian school and frequency of drinking or eating in animal handling areas. A significant interaction was found (*X* ^2^(6) = 14.46, p = 0.025), indicating that veterinarians having earned their degree earlier in time were less likely to frequently eat and drink in animal handling areas. To illustrate this trend, the 47/76 veterinarians (61.8%) obtaining their degree in 2010 or later were more likely to eat and drink in handling areas on a daily basis than the 18/54 individuals (33.3%) that graduated in 1989 or earlier. Also supporting this pattern, the 15/54 individuals (27.8%) who graduated in 1989 or earlier were also more likely to never eat and drink in handling areas while only 7/76 (9.2%) who obtained their degree in 2010 or later stated the same. An additional Chi-square test comparing this same habit against responses about length of practice (categorised as 5 years or less, 6–15 years, and more than 15 years) also provided evidence for a significant relationship (*X* ^2^(4) = 9.67, p = 0.046), demonstrating a similar trend in which those with less experience were more likely to eat and drink in handling areas. This is illustrated by the fact that 41/108 respondents (38.0%) in practice for more than 15 years were less likely to eat and drink in handling areas than the 27/45 respondents (60.0%) practicing for at most 5 years. Additionally, those 7/45 respondents (15.6%) practicing for at most 5 years were less likely to never eat and drink in handling areas than those 24/108 (22.2%) in practice for more than 15 years. Since these data were self-reported, there may be potential bias in the responses given.

Zoonotic disease reporting

As far as the reporting of zoonotic disease and infections, 199/215 (92.6%) respondents indicated they had diagnosed zoonotic disease in animals in the past 5 years (see Table 5). The 10 most frequently diagnosed zoonotic diseases in animals in order of most frequent to least frequent were: ringworm / dermatophytosis, leptospirosis, roundworms, *Giardia* infection, hookworms, Lyme disease, scabies, salmonellosis, toxoplasmosis, and Methicillin-resistant *Staphylococcus aureus* (MRSA). 60/214 (28%) respondents indicated they had been infected with a zoonotic disease in practice, with 10 respondents indicating that they had had > 1 zoonotic disease. A total of 66 cases of zoonotic disease, of which 36/65 (55.4%) were not medically confirmed, were listed by these 60 veterinarians (see Table 6). The most common zoonotic disease that was reported was dermatophytosis (also referred to as microsporum or ringworm) (n = 34), but only 14/72 (19.4%) cases were reportedly medically confirmed. When conducting a Chi-square test between years in practice and whether respondents had been infected with a zoonotic disease at their workplace, no significant relationship was found (*X* ^2^(3) = 1.61, p = 0.447). Additionally, similar insignificant results were found when running Chi-square tests between contracting zoonotic diseases and respondent’s year of graduation, sex, knowledge about the One Health initiative, having written infection control guidelines, and eating and drinking in animal handling areas. Low respondent counts limited the ability to statistically investigate potential relationships between contracting zoonotic diseases in the workplace with cleaning tables and / or frequency of handwashing.

**Table 5:** Number of diagnosed cases of zoonotic disease in animals within the past 5 years as reported by veterinarians in Michigan.

Diagnosed cases	No. of respondents	%
More than 10	133	61.9%
2–5	32	14.9%
6–10	26	12.1%
None	16	7.4%
One	8	3.7%
Total	215	100%
Diagnosed cases	No. of respondents	%

**Table 6:** Zoonotic diseases reported by veterinarians in Michigan.

Disease or condition	No. of cases	Medically confirmed	
		No.	%
Ringworm / dermatophytosis / microsporum	34	14	41%
Cryptosporidiosis	6	1	17%
Cat scratch disease / bartonellosis	5	3	60%
Scabies	4	1	25%
Salmonellosis	3	2	67%
Campylobacteriosis	2	2	100%
Giardiasis	2	1	50%
Leptospirosis	2	0	0%
Abscess	1	1	100%
Ehrlichiosis	1	1	100%
Enteritis	1	0	0%
Erysipelas	1	0	0%
Orf	1	1	100%
Psitticosis	1	1	100%
Strep	1	1	100%
Toxoplasmosis	1	1	100%
Total	66	30	45%

One Health Considerations

A number of individual factors were examined for potential relationships with knowledge about the One Health initiative. A Chi-square test assessing the association between respondents’ knowledge about the One Health initiative and length of practice demonstrated a significant relationship (*X* ^2^(2) = 16.28, p < 0.001). Although all individuals practicing 5 years or less were familiar with this concept, 31/108 (28.7%) of veterinarians practicing more than 15 years had no knowledge about the initiative. Additionally, the year of veterinary degree completion was also statistically significant (*X* ^2^(3) = 15.89, p = 0.001). A total of 71/76 (93.4%) of those graduating in 2010 or later were familiar with the initiative, and this familiarity decreased to 37/54 (68.5%) among individuals graduating in 1989 or earlier. Attending a continuing education (CE) lecture or completing CE credits on zoonotic diseases in the past 3 years was also found to be related to knowledge about One Health (*X* ^2^ (1) = 4.75, p < 0.05), such that, while 115/137 (83.9%) of respondents partaking in this educational opportunity were familiar with One Health, 40/57 (70.2%) of those not partaking in such an activity were familiar with the initiative. Sex also appeared to have a relationship with familiarity with the One Health initiative (*X* ^2^(1) = 3.92, p = 0.048). While 127/154 (82.5%) of women were aware of One Health, familiarity decreased more than 12 percentage points (41/59 [69.5%]) among men. This may have been due to the fact that women comprised a vast majority 61/76 (80.3%) of respondents who completed their degree in 2010 or later as compared to those earning their degree 1989 or earlier 23/54 (42.6%).

185/215 (86%) respondents indicated they knew who to contact if a potential case or outbreak of zoonotic disease was suspected. However, 99/215 (46.1%) respondents indicated there were ways that public health agencies could better assist with issues involving zoonotic diseases and an additional 95/215 (44.2%) were unsure if there were ways that public health agencies could better assist with issues involving zoonotic diseases. The four ways most commonly listed in order of frequency were: maintaining a website with information for veterinarians on zoonotic diseases, providing written information for clients on awareness and prevention of zoonotic diseases, providing written information about local regulations (e.g. rabies laws) and notifiable diseases, and providing written information for veterinarians on recognition and control of zoonotic diseases (see Table 7). The other suggestions respondents gave for ways that public health agencies could better assist with issues involving zoonotic diseases included:

- ‘Better authority to enforce needs for protecting from zoonotic disease (no one enforced the legal need for euthanasia of a rabies suspect, neurologic unvaccinated cat). I was left with an order to euthanise from the health department and no backup to make it happen.’
- ‘I've noticed over my practice life (25 years) that the public seems to be more uninformed about rabies with time. People really don't know anything about it, don't understand how deadly it is – since our public health system is likely irretrievably broken, not sure how to mount an educational campaign that would effectively teach people about it, but I do find the ignorance pretty frightening.’
- ‘More consistency in local regulations and local interpretation of state law. For instance, I am near border of three counties and in cases of bites by owned pets, every county has a different interpretation of state guidelines as to when rabies testing is recommended vs when home quarantine or quarantine at animal control is the allowable option. Makes it difficult as a practitioner because clients get conflicting recommendations.’
- ‘More money for testing animals for rabies.’
- ‘Public health addressing the public in general. Talking to people about the importance of getting their animals vaccinated, tested and preventatives. If all health care workers discussed with people the importance of these things then veterinarians would have greater compliance and not be looked as the bad guy just looking for money.’
- ‘Television – mail flyers.’

**Table 7:** Suggestions from Michigan veterinarians of ways public health could better assist with zoonoses awareness and prevention.

ould better assist veterinarians in dealing with zoonotic disease	No. selected	%
Website with zoonotic disease information for veterinarians	86	86.9%
Written information for clients on zoonotic disease awareness and prevention	76	76.8%
Written information about local regulations (e.g. rabies laws) and reportable diseases	75	75.8%
Written information for veterinarians on zoonotic disease recognition and control	73	73.7%
Meetings or CE on zoonotic disease held by the public health department	72	72.7%
Easier access to specific individuals at the public health department for zoonotic disease consultations	47	47.5%
On-site educational presentations or training for veterinarians and staff	42	42.4%
Recorded hotline messages about current zoonotic disease concerns	27	27.3%
Other*	7	7.1%
Total	99	

*Other suggestions are provided in the text of the article.

DISCUSSION

The objective of this study was to determine the extent to which practicing veterinarians in Michigan engaged in commonly recommended practices for the prevention of zoonotic diseases. The present study found that, of those responding, most 161/214 (75.2%) agreed that it was very important for veterinarians to educate clients on prevention of zoonotic diseases; however, relatively few 77/214 (36%) initiated discussions about zoonotic diseases with clients on a daily basis. Additionally, only 137/214 respondents (64%) indicated that they had client education materials on zoonotic diseases available in their practices. These findings are similar to those of Lipton et al. (2008) where only 203/356 (57%) of those veterinarians surveyed indicated that they had client education materials on zoonotic diseases available in their practices. Stull et al. (2012) found that of pet owners who had taken their pet to a veterinarian in the past year, only 27% reported having ever received information regarding zoonotic diseases.

Veterinarians, health care professionals (e.g. physicians, physician assistants, nurse practitioners, nurses), public health professionals, and wildlife biologists have necessary roles to play in the prevention of zoonotic diseases and have contact with the public in different settings and for different reasons. The One Health initiative stresses communication, coordination, and collaboration among human, animal, environmental health, and other relevant partners (Centers for Disease Control and Prevention, 2020b) in order to decrease the incidence of zoonotic diseases. Respondents to this survey indicated an interest in increasing communication between human and veterinary medicine and a desire for increased assistance from public health agencies regarding zoonotic disease prevention. In this survey of Michigan veterinarians, 99/215 (46.1%) of respondents indicated there were ways that public health agencies could better assist with issues involving zoonotic diseases. In protecting the public’s health from zoonoses, the authors believe a One Health approach should be taken. The One Health concept is an integrative effort of multiple disciplines (human, animal, and environmental health) working locally, nationally, and globally to achieve optimal health for people, animals, and the environment (Ryu et al., 2017).

Furthermore, the authors believe local public health agencies should take the initiative to begin developing a sustainable collaborative relationship with community veterinarians. This relationship could have four immediate goals:

1. Create a One Health Community Infrastructure;
2. Form a community One Health Team;
3. Develop a community zoonotic disease website that would be a resource to health care professionals, veterinarians, public health professionals, state and / or federal wildlife management agencies, and the community; and
4. Create and distribute to all community veterinarians and health care workers zoonotic disease information handouts that they can share with patients / clients about the prevention, detection, treatment and elimination of zoonotic disease in the community.

One campaign that One Health Team members could use to build and strengthen their interdisciplinary collaborations may be the annual One Health Day. This day was initiated in 2016 by the One Health Commission, the One Health Platform, and the One Health Initiative Team. International One Health Day is officially celebrated around the world every year on November 3 (One Health Commission, 2020). This could be a day on which an annual community education seminar (CEU) about zoonotic disease is offered to people involved in health care, animal control, public health, local government and public education. The One Health Team could also assist in creating / promoting public service announcements (PSAs) about zoonotic disease to be shared in the community by way of broadcast and social media.

Over a multiyear timeline, public health agencies working with the One Health Team could develop infection control guidelines for the community. Additionally, there should be a system in place for the local government to enforce infection control guidelines for the community. These guidelines should be congruent with state and national guidelines. In addition, veterinarians should have infection control guidelines for all staff members and the public in their practice (The National Association of State Public Health Veterinarians Veterinary Infection Control Committee, 2015; The National Association of State Public Health Veterinarians Animal Contact Compendium Committee, 2017).

Zoonotic diseases may receive more attention in the future because of coronavirus disease (COVID-19). According to the World Health Organization (2020), ‘This infectious disease caused by a newly discovered coronavirus has become a pandemic. Currently, the source of SARS-CoV-2, the coronavirus (CoV) causing COVID-19 is unknown. All available evidence suggests that SARS-CoV-2 has a natural animal origin and is not a constructed virus. SARS-CoV-2 virus most probably has its ecological reservoir in bats SARS-CoV-2 belongs to a group of genetically related viruses, which also include SARS-CoV and a number of other CoVs isolated from bat populations. MERS-CoV also belongs to this group, but is less closely related.’ Veterinarians and public health professionals should seize the opportunity of the current focus on zoonotic conditions to be included in decisions that are made at the local, state, and national level in regard to these infectious diseases.

This study was able to capture the strong support and desire among veterinarians to collaborate with health professionals. The following comments corroborate the aforementioned discussion by demonstrating a need for increased communication between veterinarians and physicians (cornerstones to One Health):

- ‘Human health care needs to help veterinary health by supporting the importance of vaccines, preventative and testing.’
- ‘Human medical personnel tend to be vastly ignorant of zoonotic diseases.’
- ‘I feel I talk about parasites with families with puppies and children almost every time. And we discuss Lepto daily. But fail at other zoonotic diseases. I state that usually they never hear anything from their doctor or pediatrician and they always confirm it. It seems in my area a lot of ignorance or not taking the time to discuss these things from human health care.’
- ‘I feel that human health professionals (MDs, DOs, etc.) lack zoonotic disease familiarity.’
- ‘I think this is a great survey that might bring to light just how little the human medical world knows and educates their clients about diseases they can give to or get from their pets.’
- ‘Medical professionals do not appear to be receptive to our recommendations and often give differing medical advice than I would recommend. In realising the differences (and understandable confusion by clients) I’ve reached out to discuss it with them and have been met with either no response (won’t return calls) or blatant disrespect for our profession’s knowledge. I’ve reached out to three different MDs, two never returned my calls even with repeated attempts, and the one that did not respect our profession’s knowledge of zoonotic transmission.’
- ‘I think it would be helpful to educate MDs on this topic, vets realise they know more than MD on this.’

It appears that, among the respondents, the amount of time practicing as a veterinarian does not expose respondents to zoonotic diseases. Of the zoonotic diseases most frequently transmitted to veterinarians in this study, ringworm was the most frequently reported and medically confirmed (see Table 6). Ringworm was also the most frequently reported zoonotic disease diagnosed in animals in this study. Ringworm is a fungal dermatologic (skin disease), treatable with antifungal medications. There is no known preventative for ringworm besides avoiding contact with infected people, animals, and objects. The second most frequently diagnosed zoonotic disease in animals in this study was leptospirosis. Leptospirosis was the most commonly 178/213 (83.6%) veterinarian-initiated zoonotic disease discussion topic with clients (see Table 4). Leptospirosis has been reinstated as a U.S. nationally notifiable condition as of January 2013 and the incidence rate per 100,000 increased from 0.03 to 0.04 during 2016–2018 (Centers for Disease Control and Prevention, 2021). Since clients only initiated discussions about this topic 3.3% of the time in the present study, this finding underscores the need for public education about zoonotic diseases. Dogs can be vaccinated annually to prevent many strains of leptospirosis infection, but clients may not be aware or understand the implications of vaccination.

Additional survey respondent comments underscore the need for public education about zoonotic diseases:

- ‘I appreciate this survey because I think more work should be done to educate the public about all of the different zoonotic diseases.’
- ‘The present Corona virus issue we are dealing with makes us all hyper-vigilant about the possibility of disease transmission and what we are able to do to minimise the risk.’
- ‘There is too much responsibility on veterinarians to educate the general public about zoonotic disease. It needs to start with public awareness, adoption facilities, etc.’
- ‘There should be state level phone helpline just like Poison Control help line.’
- ‘I would like the state to send out a monthly report of all cases reported and summarising regions where they were diagnosed. I will follow-up personally but very important sort and unfortunately neglected.’
- ‘This is an important topic and I am glad you are studying it. I think as the older generation of practitioners who in my experience don't seem to be that concerned about public health in day practice phases out it is a great opportunity to make public health more visible in private practice where we can make a big impact on how our clients interact with their pets and other animals.’

The authors suggest that veterinarians make available attractive, easily read, client educational materials that discuss common zoonotic diseases transmissible from pets and offer practical and effective advice for control. The Centers for Disease Control and Prevention maintains several webpages regarding zoonotic diseases and their prevention, including ‘Healthy Pets, Healthy People’, which has links to many client educational resources, such as brochures and posters (Centers for Disease Control and Prevention, 2020a).

This study had several limitations. The response rate being 402/3410 (11.8%) may have contributed to non-response bias, which may have skewed gender and other differences in results. This bias in response rate may have been due to the time constraint involved. The veterinarians were given less than 30 days to respond to the survey; more time may have resulted in a better response rate. The survey was electronic and not all respondents answered all questions. Hohwü et al. (2013) wrote that response rates in most studies have been reported to be lower in web-based questionnaires than in paper-based questionnaires. Perhaps a paper survey would have received a better response rate and completion rate. The declining response rates in population-based surveys in general are a challenge to epidemiology (Hohwü et al., 2013). The survey was sent via email (obtained from Michigan LARA), some of which were personal emails, leading to at least three replies the authors perceived as negative feedback. This survey did not have promotion from an organisation like a veterinary medical association or public health institution, which could have increased the response rate. This study took place in March 2020, during which a United States National Emergency was announced and the spread of the SARS-CoV2 virus would eventually be classified as a pandemic. This may have decreased the participation of veterinarians in this survey. The survey question asking for the type of practice allowed respondents to choose more than one option, as well as write in options which made analysis of this question difficult. Respondents were allowed to write in their veterinary degree granting institution and some chose to provide acronyms that the author could not confirm.

Overall, this study found that veterinarians in the state of Michigan understood the importance of preventative measures and wished to further work alongside health professionals in human health to prevent (and / or treat) zoonotic diseases. One initiative dedicated to doing this is the One Health campaign, with which recent veterinary graduates (since 2010) were more familiar. Veterinarians expressed concern that human medical education also needs to stress the importance of One Health and the communication between veterinary and human medical fields. Additionally, many recent veterinary school graduates reported not practicing zoonotic disease prevention practices, such as not eating / drinking in animal handling areas, which may need to be stressed in veterinary medical education. There also appears to be a need for public health agencies to increase their communication with, and support of, veterinarians in the effort to prevent zoonotic disease transmission including public education on this topic. Nevertheless, veterinarians acknowledge the need and express willingness to cooperate in a multidisciplinary approach to addressing zoonotic illnesses. With the third COVID-19 wave gaining momentum at the time of this writing, this study points to encouraging signs for the future in establishing concerted efforts in preventing and treating zoonoses, with veterinarians as key players.

Footnotes

1. Survey instrument available from the corresponding author.
2. Qualtrics, version March 2020, Provo, UT.
3. SPSS, version 25.0, SPSS Inc, Armonk, NY.

## Conflict of Interest

The author declares no conflicts of interest.

**Acknowledgements:** We thank C. Chen, Central Michigan University Statistical Consulting Center, for providing an independent review of our study to determine that our findings and conclusions were appropriate given the sample design and sample size and potential biases. Thank you to Kaylan Fitch, MPH, for the technical assistance with Qualtrics software.

Supplementary files

Fig 1. Practice Type – Surveyed Michigan Veterinarians

Fig 2. Zoonotic disease discussion frequency

Fig 3. Percent of surveyed Michigan veterinarians who diagnosed cases of zoonotic disease in animals

## Disclaimer

Knowledge Summaries are a peer-reviewed article type which aims to answer a clinical question based on the best available current evidence. It does not override the responsibility of the practitioner. Informed decisions should be made by considering such factors as individual clinical expertise and judgement along with patient’s circumstances and owners’ values. Knowledge Summaries are a resource to help inform and any opinions expressed within the Knowledge Summaries are the author’s own and do not necessarily reflect the view of the RCVS Knowledge. Authors are responsible for the accuracy of the content. While the Editor and Publisher believe that all content herein are in accord with current recommendations and practice at the time of publication, they accept no legal responsibility for any errors or omissions, and make no warranty, express or implied, with respect to material contained within. For further information please refer to our Terms of Use.
